# Machine Learning Approaches for MDD Detection and Emotion Decoding Using EEG Signals

**DOI:** 10.3389/fnhum.2020.00284

**Published:** 2020-09-23

**Authors:** Lijuan Duan, Huifeng Duan, Yuanhua Qiao, Sha Sha, Shunai Qi, Xiaolong Zhang, Juan Huang, Xiaohan Huang, Changming Wang

**Affiliations:** ^1^Faculty of Information Technology, Beijing University of Technology, Beijing, China; ^2^Beijing Key Laboratory of Trusted Computing, Beijing, China; ^3^National Engineering Laboratory for Critical Technologies of Information Security Classified Protection, Beijing, China; ^4^College of Applied Sciences, Beijing University of Technology, Beijing, China; ^5^Beijing Anding Hospital, Capital Medical University, Beijing, China; ^6^Advanced Innovation Center for Human Brain Protection, Capital Medical University, Beijing, China; ^7^Brain-inspired Intelligence and Clinical Translational Research Center, Xuanwu Hospital, Capitap Medical University, Beijing, China; ^8^Department of Neurosurgery, Xuanwu Hospital, Capitap Medical University, Beijing, China

**Keywords:** EEG, major depressive disorder (MDD), interhemispheric asymmetry, cross correlation, feature

## Abstract

Emotional decoding and automatic identification of major depressive disorder (MDD) are helpful for the timely diagnosis of the disease. Electroencephalography (EEG) is sensitive to changes in the functional state of the human brain, showing its potential to help doctors diagnose MDD. In this paper, an approach for identifying MDD by fusing interhemispheric asymmetry and cross-correlation with EEG signals is proposed and tested on 32 subjects [16 patients with MDD and 16 healthy controls (HCs)]. First, the structural features and connectivity features of the θ-, α-, and β-frequency bands are extracted on the preprocessed and segmented EEG signals. Second, the structural feature matrix of the θ-, α-, and β-frequency bands are added to and subtracted from the connectivity feature matrix to obtain mixed features. Finally, the structural features, connectivity features, and the mixed features are fed to three classifiers to select suitable features for the classification, and it is found that our mode achieves the best classification results using the mixed features. The results are also compared with those from some state-of-the-art methods, and we achieved an accuracy of 94.13%, a sensitivity of 95.74%, a specificity of 93.52%, and an F1-score (f1) of 95.62% on the data from Beijing Anding Hospital, Capital Medical University. The study could be generalized to develop a system that may be helpful in clinical purposes.

## Introduction

Major depressive disorder (MDD) is a major mental disorder and is characterized by loss of interest, poor concentration, and even suicidal thoughts (Acharya et al., 2018).

It has been reported that more than 264 million people worldwide suffer from depression, which heavily impacts quality of life (World Health Organization, 2020). An accurate diagnosis of MDD is of great importance for early intervention and effective treatment. Traditional diagnosis of MDD mainly depends on subjective evaluation of symptom intensity using interview sessions and psychiatric scales. These methods are useful but time consuming and sometimes may lead to misdiagnoses due to human and environmental factors. Thus, it is crucial to develop objective approaches to help clinicians diagnose MDD more effectively.

Electroencephalography (EEG) is a noninvasive technique with high temporal resolution; this technique is sensitive to changes in the functional state of the human brain (Schmidt et al., 2013). Resting-state EEG (rsEEG) reveals brain network activity and can be applied to neurological evaluations (Tóth et al., 2014). EEG signals can be viewed as a group of multivariate time series, and extracting features is essential to tracking changes in EEG signals (Ting et al., 2008). Studies of depression have found that depressed patients show significant obstacles in interpreting fear, anger, happiness, surprise, and sadness (Filomena et al., 2016). Depressed patients are different from healthy subjects in the decoding of negative emotions. In a study, it was found that electroconvulsive therapy (ECT) could modulate the functional connectivity of the left angular gyrus in patients with depression (Wei et al., 2018). From the performance and treatment of depression, it can be concluded that there may be differences in brain structure between patients with depression and healthy subjects. Various studies indicate that interhemispheric frontal EEG α asymmetry is considered a key marker of structural alteration of the human brain in MDD (Allen et al., 2004; Allen and Reznik, 2015; Cantisani et al., 2015; Mumtaz et al., 2017a). Except for the α frequency band, activity in other bands and brain regions may also be associated with a disordered brain state caused by MDD, and EEG signals confounded with noises also influence the identification of specific signals. It has also been investigated whether brain connectivity is altered in MDD patients (Iseger et al., 2017). Therefore, connectivity should be taken into consideration in the recognition of special EEG signals. In real EEG data classification tasks, extracting reliable EEG features is sometimes challenging, and EEG signals in depression have both structural (Michalopoulos and Bourbakis, 2015) and connectivity features. Therefore, we propose a mixture of structural features and connectivity features for MDD classification; that is, we extract features from different viewpoints and combine them together for MDD classification.

In recent years, as the main type of artificial intelligence, deep learning (DL) has been widely used for the classification and prediction of patterns in EEG signals. DL methods can extract many abstract features from a large set of training data without human supervision. In this paper, we utilize the K-nearest neighbor (KNN) (Dasarathy, 1997), support vector machine (SVM) (Cortes, 1995) and convolutional neural network (CNN) algorithms to verify the effectiveness of the extracted features for the classification of EEG signals for patients with MDD and healthy controls (HCs).

In the literature, various features have been extracted from EEG signals and have shown the importance of MDD diagnosis. Mantri et al. (2015) reported a classification accuracy of 84% based on the power spectrum, involving 13 patients with depression and 12 HCs. In 2017, Mumtaz et al. (2017b) extracted features using wavelet transform to achieve an accuracy of 87.5%. Acharya et al. (2018) attained a high accuracy of 94% from the left hemisphere and 96% from the right hemisphere. Despite all of these research findings, the clinical applications of structural features and connectivity features remain largely unclear.

In this paper, two types of features, including the interhemispheric asymmetry value and cross-correlation value, are extracted from segmented EEG epochs, and the extracted structural and connectivity changes are combined using addition and subtraction rules for the classification. Several classifiers are introduced to verify the effectiveness of the extracted features and achieve emotion decoding.

The paper is organized as follows: in section materials and methods, the dataset is described, preprocessing is performed, and the main framework of the proposed approach is given. In section results, the experimental results are given; the conclusion and discussion are presented in section discussion and section conclusion, respectively.

## Materials and Methods

### Participants and Criteria

In this study, experimental data were acquired from 32 subjects (16 patients with MDD and 16 HCs) recruited from Beijing Anding Hospital, Capital Medical University. The experiment was approved by the Ethics Committee of Beijing Anding Hospital, Capital Medical University. All the participants signed consent forms for participation and were fully informed of the experimental and data acquisition procedures. The inclusion and exclusion criteria are based on the symptoms of depression as mentioned in the section in the Diagnostic and Statistical Manual of Mental Disorders (DSM-IV) on depression (Hu, 2003). MDD participants with psychotic symptoms, pregnant patients, people with alcoholism and patients with epilepsy were excluded. The HCs were screened for possible mental or physical illness and were found to be disease free.

Independent samples *t*-test was used to measure the difference in demographic and neuropsychological assessments between the MDD and HC groups; the analysis was performed in SPSS 20.0 (IBM SPSS, Inc., Armonk, NY, USA). The significance level was set to *p* < 0.05. The results are shown in Table 1. In the descriptive analysis of the demographics, the two groups are matched in age, sex, and education level.

**Table 1:** Demographic and clinical information.

**Factors**	**MDD**	**HC**
Age (years)	31.0 ± 1.0	26.1 ± 5.4
Sex (male/female)	7/9	7/9
Education (years)	12.5 ± 1.0	13.0 ± 2.6
HAMD	19.3 ± 8.9	-

### Recording and Preprocessing of EEG Signals

**Figure 1:**
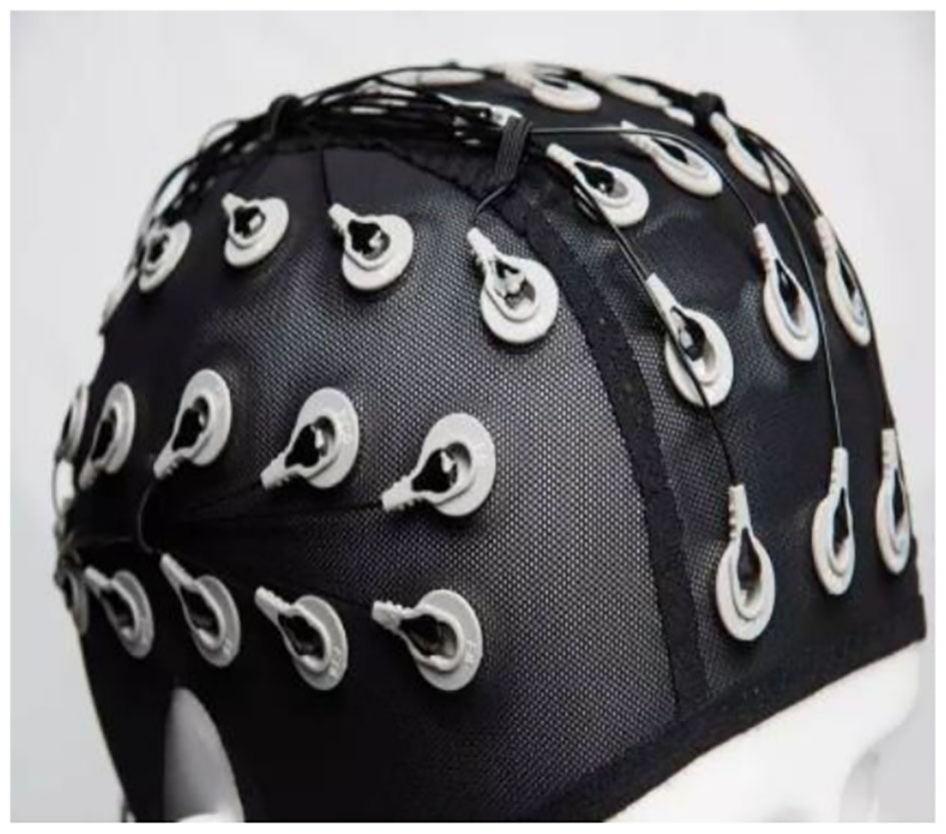
The EEG headset used to collect the data.

The rsEEG signal recordings were performed in Beijing Anding Hospital, Capital Medical University. During the EEG recording period, all the subjects sat in a comfortable armchair, were relaxed and stayed awake for about 3 min in a quiet, dim room, with room temperature maintained at 23 ± 2°C. The EEG headset used to collect the data is shown in Figure 1.

**Figure 2:**
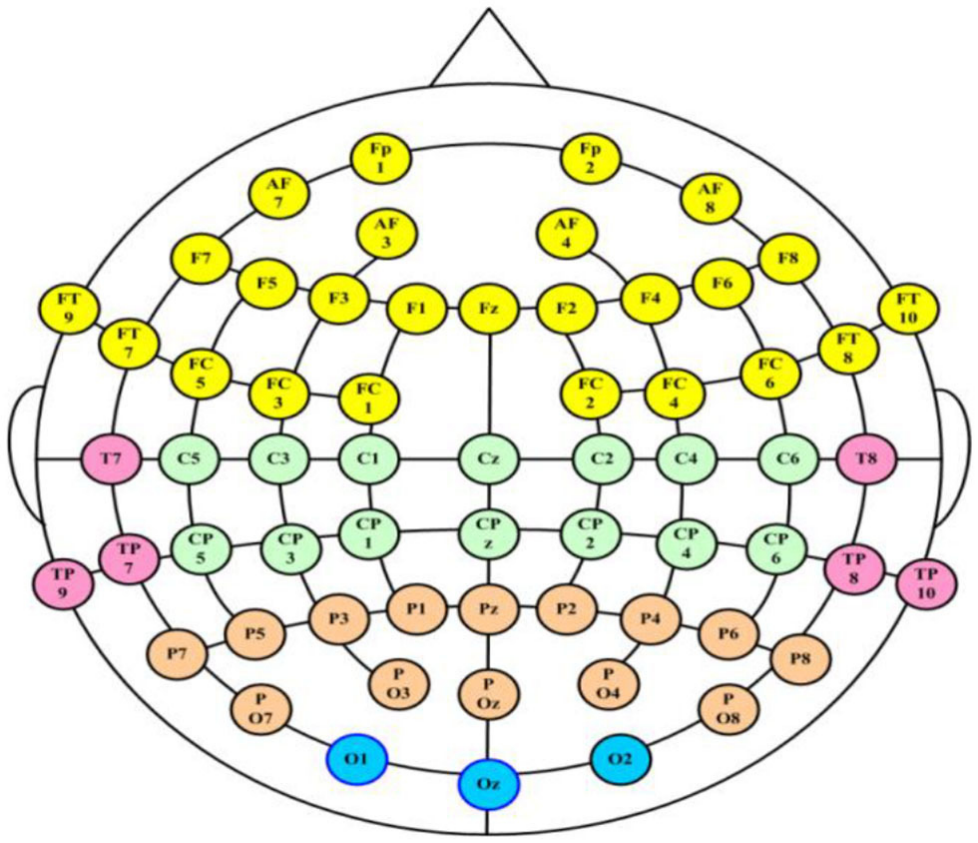
The distribution of the electrodes in the acquisition system.

The 3-min rsEEG data were recorded from 64-channel brain products with the averaged mastoids (M1 and M2) as the reference electrodes. The channel location is shown in Figure 2. The EEG data were collected with electrode impedances below 10 kΩ.

### Framework

**Figure 3:**
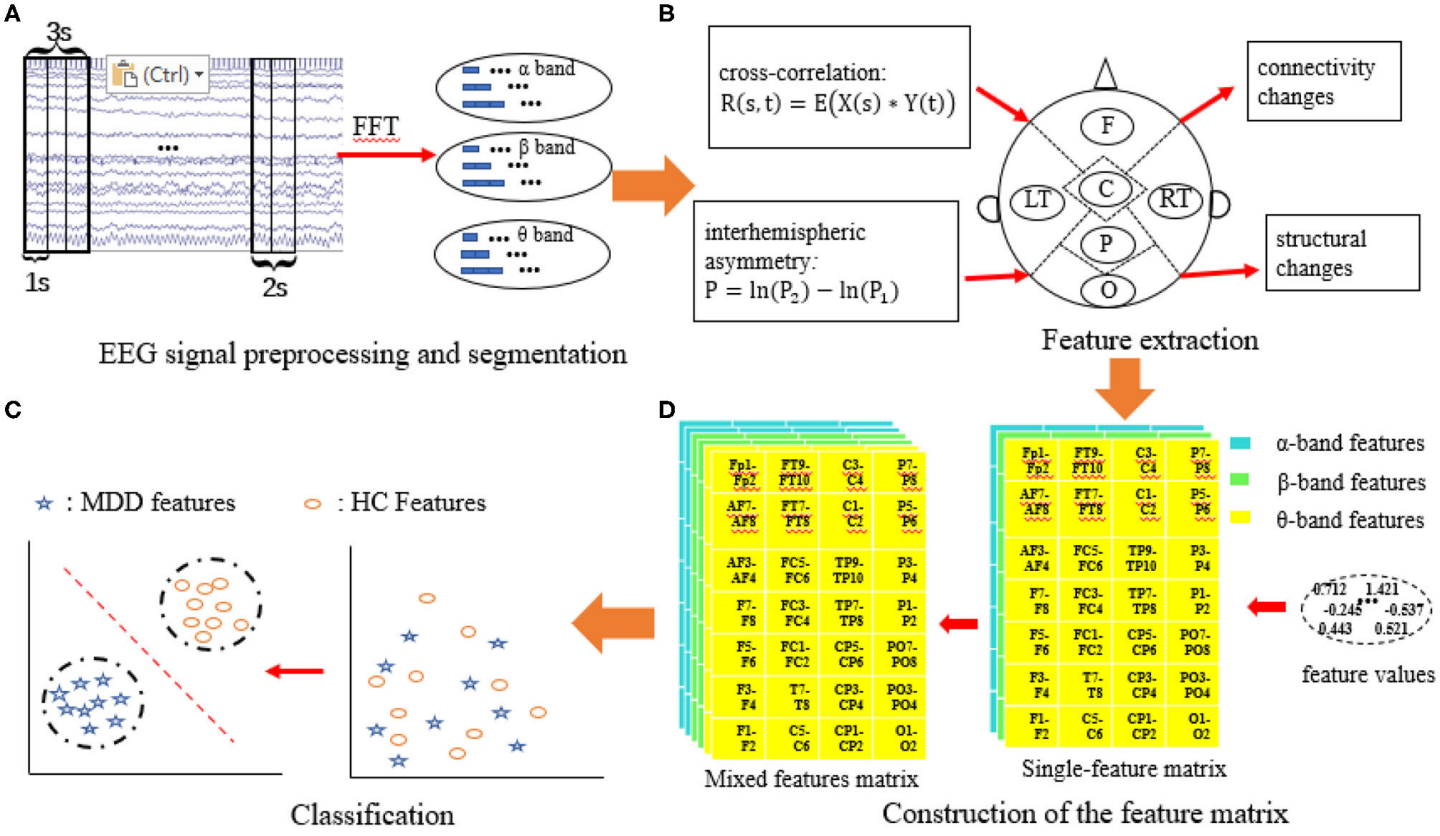
MDD EEG analysis framework. **(A)** EEG signal preprocessing and segmentation; **(B)** Feature extraction; **(C)** Construction of the feature matrix; **(D)** Classification.

The MDD EEG analysis framework is shown in Figure 3, and it mainly contains four parts: (1) EEG signal preprocessing and segmentation; (2) feature extraction; (3) construction of the feature matrix; and (4) classification.

#### EEG Signal Preprocessing and Segmentation

To comprehensively analyze the changes in patients with MDD, 28 pairs of electrodes from five brain regions (the frontal region, temporal region, central region, parietal region and occipital region) and three frequency bands [the θ-frequency band (4–8 Hz), the α-frequency band (8–13 Hz), and the β-frequency band (13–40 Hz)] were used to conduct experiments to explore the changes in interhemispheric asymmetry in MDD patients.

In this study, the recorded EEG data have a high temporal sensitivity and are extremely susceptible to external interference during collection. For example, eye blinks, movements and muscular activates (e.g., the heart beats) could cause EEG artifacts, and the EEG data with these artifacts may not truly represent the underlying brain activities. Hence, removing artifacts is an essential preprocessing step for further data analysis. We used a finite impulse response (FIR) filter to filter out unnecessary signals, and frequencies of 0.5–47 Hz remained for the analysis. Then, the independent component analysis (ICA) algorithm in EEGLAB was applied to remove ocular artifacts from the raw EEG data (Delorme and Makeig, 2004).

EEG signals are time-varying and nonstationary signals. There are different frequency components at different times and in different states. As machine learning techniques require a large number of training sets, we divided each channel in the EEG data into small, non-overlapping segments with durations of 1s, 2s, and 3s. Thus, we have a large number of samples to avoid underfitting. The sample information is given in Table 2. The average EEG recording time for all subjects is 3 min; however, this time was not the same for all the patients, so the number of epochs in the MDD and HC groups are slightly different. Three different frequency bands of EEG data, θ (4–8 Hz), α (8–13 Hz), and β (13–40 Hz), are extracted from the segmented EEG signals using a fast Fourier transform (FFT), and the number of FFT points is set to 1,024. Welch's method is applied to calculate the power spectrum of EEG bands. Welch's method consists of splitting the time series signal into epochs, computing a modified periodogram for each epoch, and then averaging the power spectrum density estimates (Alkan and Kiymik, 2007).

**Table 2:** Basic information on the samples.

**Time window size (s)**	**1**		**2**		**3**	
**Sample label**	**MDD**	**HC**	**MDD**	**HC**	**MDD**	**HC**
Number of samples	20,143	16,708	10,068	8,349	5,031	4,172

#### Feature Extraction

Two EEG features, namely, the interhemispheric asymmetry and cross-correlation, are extracted. Then, the two features are combined in two ways.

##### Interhemispheric asymmetry

The interhemispheric asymmetry is computed by the power value of the electrode in the left and right brain regions. The interhemispheric EEG asymmetry is shown in **Equation (1)**:

(1) $$P=ln(P_{2})-ln(P_{1})$$

P denotes the interhemispheric asymmetry value. *P*_2_ is the power value of one electrode in the left brain region, *P*_1_ is the power value of the electrode in the right brain region, ln(*P*_2_) indicates the absolute power of the left brain region, and ln(*P*_1_) is the absolute power of the right brain region.

##### Cross-correlation

The formula for calculating the correlation coefficient of the two symmetric electrodes X(s) and Y(t) is:

(2) $$\begin{array}{r} R\left(s,t\right)\;=\;E\left(X\left(s\right)\;*Y\left(t\right)\right) \end{array}$$

where ^*^ indicates the convolution of the two sequences. The correlation coefficient is normalized by:

(3) $$\begin{array}{r} \text{R }=\;\frac{\hat{\text{R}}-\min\left(\text{R}\right)}{\max\left(\text{R}\right)-\min\left(\text{R}\right)} \end{array}$$

The range of *R* is from 0 to 1. The larger the value of the correlation is, the greater the correlation between the two electrodes.

##### Feature mixing

The features are extracted and constructed into data matrices. To avoid information loss for a single feature and to improve the classification accuracy, the EEG features are combined. Two ways of combining features are attempted to provide a better presentation of human brain state changes in MDD. To remedy the information deficiency of single features, the two single features (the feature matrix) are added together using formula (4). To reduce the amount of redundant information, the two types of features are combined using formula (5).

(4) $$\begin{array}{r} MIX1\;=\frac{k_{1}}{k_{1}+k_{2}}\;*\;F_{1}+\frac{k_{2}}{k_{1}+k_{2}}\;*\;F_{2} \end{array}$$

(5) $$\begin{array}{r} MIX2\;=\frac{k_{1}}{k_{1}+k_{2}}\;*\;F_{1}-\frac{k_{2}}{k_{1}+k_{2}}\;*\;F_{2} \end{array}$$

where k_1_ and k_2_ are the ingredient coefficients of the two features and their range is from 0 to 1; both k_1_ and k_2_ are set to 0.5. F_1_ denotes the interhemispheric asymmetry matrix, and F_2_ denotes the cross-correlation matrix. MIX1 is an index indicating the integrated brain state of interhemispheric asymmetry and cross-correlation. MIX2 is an index indicating the difference in the brain state of interhemispheric asymmetry and cross-correlation.

#### Construction of the Feature Matrix

Three feature matrices are constructed to feed into the classifiers: two are single-feature matrices, and the third is the mixed-feature matrix.

The single-feature matrix contains three layers: the first layer is α interhemispheric asymmetry (or cross-correlation), the second layer is β interhemispheric asymmetry (or cross-correlation) and the last layer is θ interhemispheric asymmetry (or cross-correlation).

**Figure 4:**
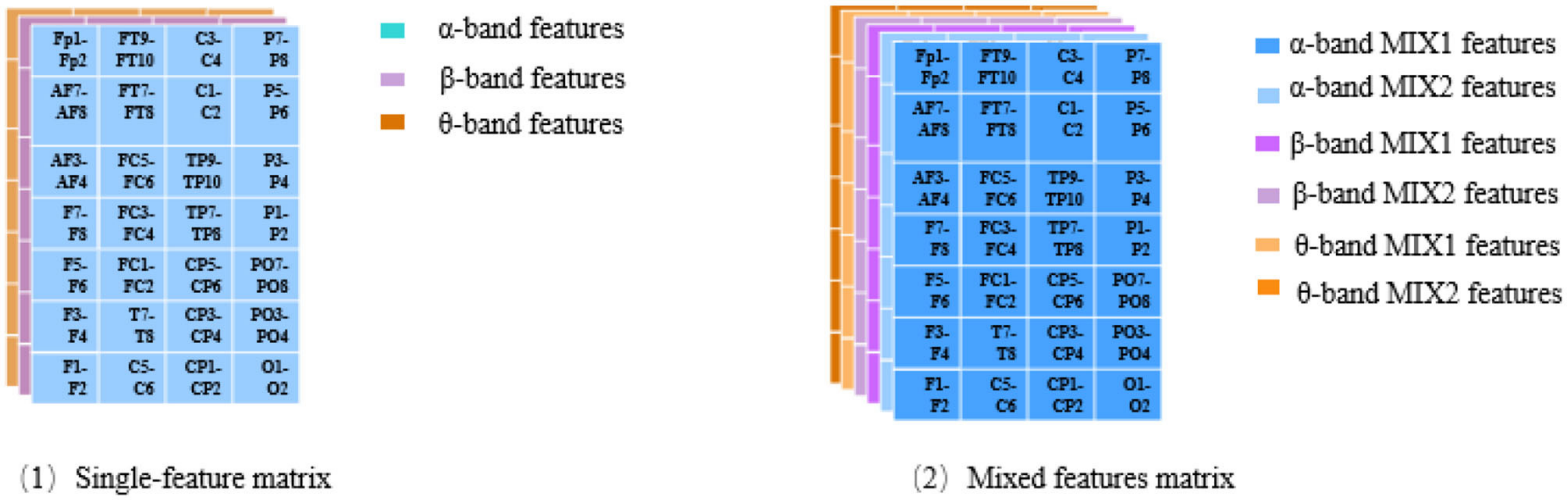
Structure of the feature matrix.

The mixed-feature matrix contains six layers: the first two layers are the MIX1 and MIX2 feature matrices in the α band, the middle two layers are the MIX1 and MIX2 feature matrices in the β band, and the last two layers are the MIX1 and MIX2 feature matrices in the θ-frequency band. Thus, the size of the single-feature input matrix is 7 × 4 × 3, while the size of the mixed-feature input matrix is 7 × 4 × 6. The structure of the single-feature matrix and mixed feature matrix are shown in Figure 4.

#### Classification

##### Classifier

Selecting a suitable classifier is important for MDD identification, and the KNN, SVM, and CNN algorithms are used to verify the effectiveness of the extracted features.

The KNN algorithm, which was proposed by Dasarathy (Dasarathy, 1997) in 1991, is a basic machine learning method used for classification and regression. It is adept at handling noise and large datasets. It performs classifications by a majority voting of the neighbors, with the case being assigned to the class most common among its K-nearest neighbors measured by a distance function. The algorithm involves three main factors: a training set, distance or similarity measure, and the size parameter K. Several distance metrics are utilized to define the distance or similarity in the KNN technique. To avoid the matching problem between objects, the Euclidean distance is used. The KNN algorithm has been widely used in EEG signal detection fields, such as epilepsy (Acharya et al., 2012), anxiety disorder (Wang et al., 2013), and depression (Rowley and Kanade, 1998). In this study, K is set to 7 to ensure a better classification accuracy.

The SVM algorithm, which was proposed by Cortes and Vapnik (Cortes, 1995) in 1995, is a supervised machine learning method used in classification and regression. The SVM algorithm can discriminate non-linearly separable data by mapping them to higher dimension space by using a kernel function to make the data more separable. We chose a poly kernel function; the degree of the polynomial is set to 3, gamma is set to 2, and the maximum number of iterations is set to 30,000.

**Figure 5:**
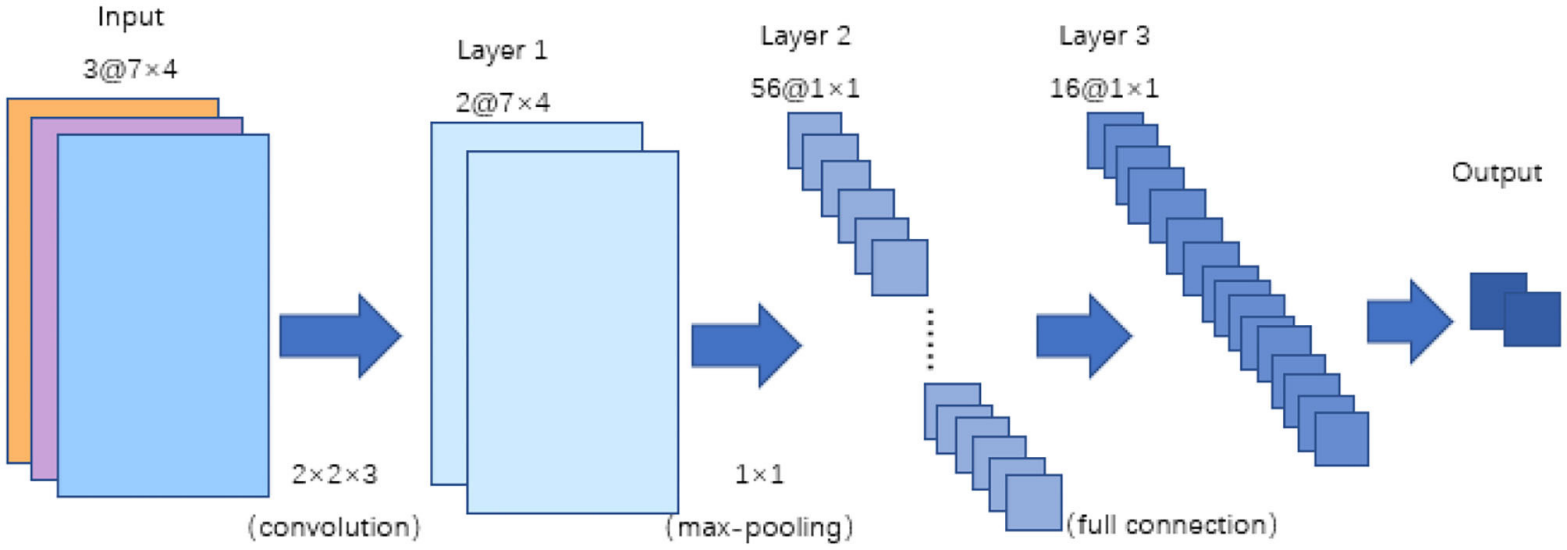
Structure of the CNN.

CNN is a kind of feedforward neural network with a deep structure and convolutional computations, and it is one of the representation algorithms of deep learning. The CNN used in this study mainly contains three layers: a convolutional layer, a pooling layer and a fully connected layer. The structure of the CNN is shown in Figure 5. As shown in the figure, in the convolutional layer, two 2 × 2 × 3 convolution kernels are selected. The outputs of the convolutional layers are two 7 × 4 × 2 feature maps, and they are the input of the pooling layer. We chose max pooling, and the step size is set to 1. After reshaping, the output matrix is resized 1 × 1 × 56, and it is input into the fully connected layer. To overcome overfitting in the fully connected layer, the dropout method is applied to each layer, and 50% of the training results are retained.

##### Evaluation of the classification performance

To evaluate the performance of different classifiers with different EEG features, the following statistical measures are utilized.

(1) Accuracy: The accuracy is defined as the percentage of correctly classified EEG segments of MDD patients and HCs, and it is defined mathematically in formula (6). False positives (FP) and false negatives (FN) are misclassifications of MDD and HC, respectively.

(6) $$\begin{array}{r} Accuracy\;=\frac{TP+TN}{TP+FN+TN+FP} \end{array}$$

where TP indicates the number of true positives, TN indicates the number of true negatives, FN indicates the number of false negatives and FP indicates the number of false positive.

(2) Sensitivity: The sensitivity is evaluated by the accuracy rate of the positive samples, and it is defined as the accuracy rate of the MDD EEG epochs and is given by formula (7).

(7) $$\begin{array}{r} Sensitivity\;=\frac{TP}{TP+FN} \end{array}$$

(3) Specificity: The specificity is defined as the accuracy rate of the negative samples. It is defined as the accuracy rate of the HC EEG epochs and is given by formula (8).

(8) $$\begin{array}{r} Specificity\;=\frac{TN}{TN+FP} \end{array}$$

(4) F1-score: The F1-score is regarded as the weighted average of the model precision and recall. It is defined by formula (9); its maximum value is 1, and its minimum value is 0.

(9) $$\begin{array}{r} F1\;-\;score\;=\frac{2\;*\;TP}{2\;*\;TP+FP+FN} \end{array}$$

## Results

To assess the ability of the proposed framework to detect and classify MDD EEG signals, several experiments are conducted, which mainly contain statistical analysis and classification. The statistical analysis was performed by one-factor analysis of variance (ANOVA) using SPSS 22.0. The classification is implemented in PyCharm (version 2017.3.4, Community Edition).

### Statistical Analysis Results

ANOVA was used to examine significant differences between the two groups (patients MDD and HCs). The significance level was set to *p* < 0.05. Single features (asymmetry, cross-correlation) and mixed features (MIX1 and MIX2) in the different frequency bands (α band, β band, and θ band) are all analyzed. In terms of EEG segmentation, a segmentation of 2s is demonstrated and analyzed in detail in this study.

#### Statistical Analysis of the Interhemispheric Asymmetry

**Figure 6:**
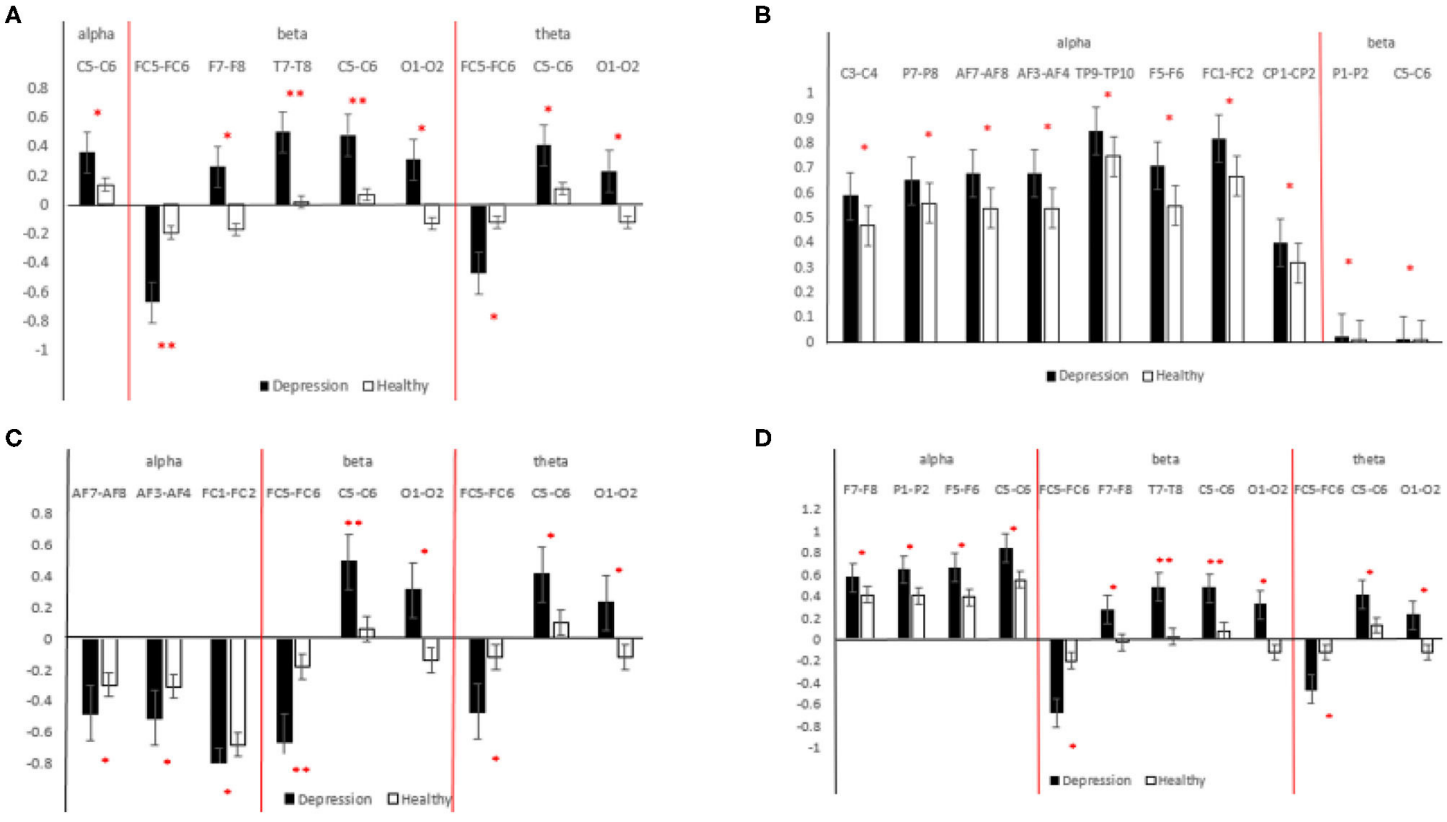
Statistical analysis results for different features [**(A)** interhemispheric asymmetry; **(B)** cross-correlation; **(C)** MIX1; **(D)** MIX2]. The black bar indicates the MDD group, and the white bar indicates the HCs. **indicates 0.001 < *p* < 0.01, *indicates 0.01 < *p* < 0.05.

The results of the statistical analysis of the interhemispheric asymmetry in all the frequency bands of the MDD and HC groups are shown in Figure 6A. A positive value for the interhemispheric asymmetry indicates that the power value of the left brain is greater than that of the right brain. Similarly, a negative value for the interhemispheric asymmetry indicates that the power value of the left brain is less than that of the right brain.

As shown in Figure 6A, in the α-frequency band, the interhemispheric asymmetry in patients with MDD at C5-C6 is significantly higher than that of the HCs. In the β-frequency band, the significant electrode pairs for the interhemispheric asymmetry are from the whole brain except the parietal region, and the values for the patients MDD are significantly larger than those of the HCs. The significant electrode pairs of the interhemispheric asymmetry are from the frontal, central and occipital regions. From Figure 6A, it is easy to see that the values for the patients with MDD are significantly larger than those of the HCs, which indicates that the difference between the interhemispheric power in patients with MDD is larger than that in HCs. The importance of EEG alpha interhemispheric asymmetry in the diagnosis of depression is evident from various studies. For example, hypo-activation of the left frontal has been observed during MDD (Kemp et al., 2010).

#### Statistical Analysis of the Cross-Correlation

The results of the statistical analysis of the cross-correlation in patients with MDD and HCs are shown in Figure 6B. The significant electrode pairs in the α-frequency band came from the whole brain except for the occipital region, and the cross-correlation in the patients with MDD was significantly larger than that in the HCs, which means that compared with the HCs, EEG connectivity in patients with MDD in the α-frequency band was enhanced. There was no significant difference in patients with MDD and HCs in terms of the cross-correlation in the θ-frequency band. The significant electrode pairs of cross-correlation in the β-frequency band came from the parietal and central regions, and the cross-correlation values for the patients with MDD were significantly larger than those of the HCs. From the results of the statistical analysis of the cross-correlation, it is easy to see that patients with MDD have more brain connectivity than HCs. EEG signals in depression have connectivity features. Knott et al. (2001) reported that significant group differences in inter-hemispheric coherence pervaded all four frequency bands.

#### Statistical Analysis of MIX1

The results of the statistical analysis of MIX1 in all the frequency bands of the patients with MDD and the HCs are shown in Figure 6C. As shown in this figure, for significant electrode pairs in all the frequency bands, the value of MIX1 in the patients with MDD is larger than that in the HCs. MIX1 indicates the integrated brain state of interhemispheric asymmetry and cross-correlation. In the α-frequency band, the significant electrode pairs are all from the frontal region. In the β-frequency band, the significant electrode pairs are from the frontal, central and occipital regions. For brain regions such as central, temporal, frontal and parietal, the depressed individual showed greater anterior EEG activity. In a study, greater left frontal activity is associated with fewer depressive symptoms (Deslandes et al., 2008).

#### Statistical Analysis of MIX2

The results of the statistical analysis of MIX2 in all the frequency bands of the patients with MDD and the HCs are shown in Figure 6D. MIX2 is an index indicating the differential brain state of the interhemispheric asymmetry and cross-correlation. As shown in Figure 6D, for significant electrode pairs in all the frequency bands, the value of MIX2 in the patients with MDD is larger than that of the HCs. The significant electrode pairs in the α-frequency band are from the frontal, central and parietal regions. The significant electrode pairs in the β- and θ-frequency bands are from the frontal, central and occipital regions. In addition to α-frequency band, activity in other bands such as θ-frequency band has shown relevance such as a decreased frontal theta activity has also been reported (Saletu et al., 2010).

### Classification Results

A 10-fold cross-validation scheme is performed to prevent overfitting. All the feature matrices are randomly divided into 10 groups, nine of which are used for training, and the other group is used for verification. To ensure the stability of the classification model, each experiment is performed 10 times, and the averaged value is considered the result. At the same time, we set the shuffle parameter in this method to shuffle the data before splitting into batches. In this way, we reduce the error rate. The interhemispheric asymmetry, cross-correlation, and mixed features of the 1s, 2s, and 3s segments of the α-, β-, and θ-frequency bands in the MDD and HC groups were analyzed. The classification results are given in Table 3. In Table 3, the standard error of the classification results is around 0.001.

**Table 3:** Classification results of the EEG signals from all classifiers.

**Classifiers**	**Feature**	**1s**				**2s**				**3s**			
		**Acc (%)**	**Sen (%)**	**Spe (%)**	**f1 (%)**	**Acc (%)**	**Sen (%)**	**Spe (%)**	**f1 (%)**	**Acc (%)**	**Sen (%)**	**Spe (%)**	**f1 (%)**
KNN	F1	79.10	86.58	70.07	81.89	81.76	88.19	74.04	84.08	80.74	87.76	72.29	83.27
	F2	62.38	71.29	51.64	67.43	59.98	68.81	49.38	65.25	81.74	83.01	80.10	82.70
	MF	79.50	87.29	70.13	82.30	83.15	88.97	76.14	85.22	82.43	88.51	75.10	84.61
SVM	F1	83.78	85.88	81.52	85.36	84.13	86.24	81.60	85.59	82.83	85.53	79.62	84.46
	F2	76.31	78.55	73.62	78.36	80.91	83.15	78.26	82.62	84.27	83.49	84.97	83.21
	MF	87.95	89.24	86.38	89.00	88.22	89.69	86.44	89.26	86.15	88.28	83.60	87.43
CNN	F1	91.10	91.45	89.42	91.62	92.70	93.72	91.27	93.52	92.11	93.62	92.23	91.64
	F2	93.14	92.41	94.17	93.61	93.07	93.25	92.24	94.45	93.31	94.43	93.27	92.87
	MF	94.10	93.61	91.69	93.82	**94.13**	**95.74**	**93.52**	**95.62**	93.58	94.74	93.72	94.81

*F1 indicates asymmetry, F2 indicates cross-correlation and MF indicates mixed features. Acc indicates accuracy; Sen indicates the sensitivity; Spe indicates the specificity; f1 indicates the F1-score. Bold values indicates the best performance*.

Table 3 presents the classification results in terms of the accuracy, sensitivity, specificity and the F1-score (f1) for the 1s, 2s, and 3s EEG epochs for each of the classifiers. Table 3 shows that the F2 (cross-correlation) and F1 (asymmetry) are more suitable for MDD detection than the mixed features. Each classification index for F1 is ~85%, while each classification index for F2 is ~70%. The results show the consistency in the performance of all the classifiers. The classification results of the KNN, SVM, and CNN models based on the mixed features are better than those of the single features.

Among all the classifiers, the CNN achieved the best performance with the mixed features for the 2s time window (accuracy = 94.13%, sensitivity = 95.74%, specificity = 93.52%, and f1 = 95.62%). For the SVM, the best classification results were achieved with the mixed features in the 2s time window (accuracy = 88.22%, sensitivity = 89.69%, specificity = 86.44%, and f1 = 89.26%). For the KNN, the best performance was achieved with the mixed features in the 2s time window (accuracy = 83.15%, sensitivity = 88.97%, specificity = 76.14%, and f1 = 89.26%). Compared with the segmentation results for the 1s and 3s EEG epochs, and the segmentations of the 2s time window achieve better classification results.

## Discussion

We attempted to discover the useful features reflecting the intrinsic changes in brain activity in depressed patients to construct an automatic system for MDD detection. Two types of feature matrices were computed for MDD detection, and three classifiers were introduced to classify the EEG data from patients with MDD and HCs. First, the feature matrix for interhemispheric asymmetry was fed to three classifiers, and we obtained the best classification accuracy of 92.70% using the CNN algorithm. Second, the feature matrix for electrode connectivity was fed to the three classifiers, and we achieved the best accuracy result of 93.31% using the CNN algorithm. Finally, the two types of features were added and subtracted to form mixed features for the classification, and the accuracy was greatly improved for the three classifiers. Therefore, we concluded that the feature-combining strategy is effective. Statistical analysis and automatic classification based on the extracted and mixed features were performed. The statistical analysis explored the difference in the patients with MDD and the HCs at the group level, while the classification method studied the EEG of patients' MDD in another way.

In this study, greater left frontal activity was associated with fewer depressive symptoms. In addition, EEG interhemispheric asymmetry was concluded to be a risk marker for MDD because the study participants with depressive symptoms showed less relative frontal activity than the HCs.

We also compared the detection results with those from other investigations; this comparison is given in Table 4, which shows that we achieved the best accuracy of 94.13% using the mixed features. In 2017, the accuracy was 91.67% using kernel eigen-filter-bank common spatial patterns (Knott et al., 2001). Compared with the accuracy of 60–80% involving 48 depressed patients and 26 HCs based on the Lep-Ziv complexity (Deslandes et al., 2008), our system was considerably improved. Of course, the comparisons may be improper as we used different datasets, but our analysis at least implies the importance of our feature extraction and mixing strategy. We will collect more subject EEGs for future investigations, as 32 subjects are not enough to validate the effectiveness of the developed system. Other nonlinear EEG features related to the human brain, such as fractal dimension and entropy, should be analyzed and introduced into the feature combination for MDD detection.

**Table 4:** Summary of previous works on EEG signal analysis for depression.

**Paper format**	**Year**	**Sample size**	**Feature(s) used**	**Analysis method**	**Accuracy**
Mantri et al. (2015)	2015	13 MDD and 12 HC	Power spectrum, FFT	ANN	84%
Akdemir (2015)	2015	53 MDD and 43 HC	EEG band power	DT	80%
Liao et al. (2017)	2017	12 MDD and 12 HC	Kernel eigen-filter-bank common spatial patterns	SVM	91.67%
Mumtaz et al. (2017b)	2017	34 MDD and 30 HC	Wavelet transform	LR	87.5%
Acharya et al. (2018)	2018	33 MDD and 30 HC	Left and right hemispheres	CNN	93.5% and 96%
Fan et al. (2005)	2019	48 HCC and 26 HC	Lep-Ziv complexity BP	ANN	60-80%
Our Study		16 MDD and 16 HC	Asymmetry, cross-correlation, mixed features	CNN	94.13%

## Conclusion

In this study, we propose a feature extraction and mixing method to try to discover the correlated characteristics describing intrinsic changes in depressed patients, and the feature extraction and classifiers are integrated to construct a system for the discrimination of MDD. Both interhemispheric asymmetry and cross-correlation were extracted to analyze the structural and connective changes in the EEG signals of MDD patients. The two features were combined in two ways to comprehensively interpret the brain state of MDD. Both features were helpful for MDD detection. The classification accuracy based on interhemispheric asymmetry was ~85% for the three classifiers, while the classification accuracy based on cross-correlation was ~70% using the three classifiers. The classification results using the mixed features were greatly improved compared with using the single features. We also found that the mixed features with a 2s time window using a CNN perform the best.

The proposed depressed patient detection system is promising for exploring the pathogenesis, early diagnosis, and intervention treatment of MDD. In future research, we will try to investigate more useful information for MDD detection and emotion decoding.

## Data Availability Statement

The datasets generated for this study are available on request to the corresponding author.

## Ethics Statement

The studies involving human participants were reviewed and approved by Ethics Committee of Beijing Anding Hospital, Capital Medical University. The patients/participants provided their written informed consent to participate in this study. Written informed consent was obtained from the individual(s).

## Author Contributions

HD and LD designed this study and revised and guided the experiment. HD and SQ wrote this manuscript and participated in the whole experiment process. XZ and YQ managed the whole experiment and analyzed the data. LD, SS and CW participated all the experiments. YQ, JH, XH and CW helped for the sampling process. All authors read and approved the final manuscript.

## Conflict of Interest

The authors declare that the research was conducted in the absence of any commercial or financial relationships that could be construed as a potential conflict of interest.
